# Adipokines and Inflammatory Markers in Acute Myocardial Infarction Patients with and without Obstructive Sleep Apnea: A Comparative Analysis

**DOI:** 10.3390/ijms241914674

**Published:** 2023-09-28

**Authors:** Ana L. Vega-Jasso, Luis M. Amezcua-Guerra, Héctor González-Pacheco, Julio Sandoval-Zárate, César A. González-Díaz, Jennifer Escobar-Alvarado, Jennifer D. Manzano-Luna, Malinalli Brianza-Padilla

**Affiliations:** 1 Master of Health Sciences, School of Medicine, Instituto Politécnico Nacional, Ciudad de México 07738, Mexico; anluveja@gmail.com; 2 Department of Immunology, Instituto Nacional de Cardiología Ignacio Chávez, Ciudad de México 14080, Mexico; lmamezcuag@gmail.com (L.M.A.-G.); sandovalzarate@prodigy.net.mx (J.S.-Z.); 3 Coronary Care Unit, Instituto Nacional de Cardiología Ignacio Chávez, Ciudad de México 14080, Mexico; hectorglezp@hotmail.com; 4 Postgraduate Studies and Research Section, School of Medicine, Instituto Politécnico Nacional, Ciudad de México 07738, Mexico; gonzalezantoni@hotmail.com; 5 Sleep Laboratory, UNAM-INC Translational Research Unit, Instituto Nacional de Cardiología Ignacio Chávez, Ciudad de México 14080, Mexico; jennifer.escobar.15@gmail.com; 6 Red MEDICI, Facultad de Estudios Superiores Iztacala, Universidad Nacional Autónoma de México, Tlalnepantla 54090, Mexico; danymanzano2@gmail.com

**Keywords:** obstructive sleep apnea, adipokines, acute myocardial infarction

## Abstract

An association has been suggested between acute myocardial infarction (AMI) and obstructive sleep apnea (OSA). Considering the role of adipose-tissue-derived inflammatory mediators (adipokines) and the shared risk factor of obesity in OSA and AMI, this study aimed to investigate the involvement of adipokines in AMI patients with and without OSA. Serum levels of adipokines and inflammatory mediators were quantified, and home respiratory polygraphy was conducted. A total of 30 AMI patients and 25 controls were included. Patients with AMI exhibited elevated levels of resistin (7.4 vs. 3.7 ng/mL), interleukin-6 (8.8 vs. 1.3 pg/mL), and endothelin-1 (3.31 vs. 1.8 pg/mL). Remarkably, AMI patients with concomitant OSA exhibited higher levels of resistin (7.1 vs. 3.7 ng/mL), interleukin-6 (8.9 vs. 1.3 pg/mL), endothelin-1 (3.2 vs. 1.8 pg/mL), creatin kinase (1430 vs. 377 U/L), creatine kinase-MB (64.6 vs. 9.7 ng/mL), and troponin T (2298 vs. 356 pg/mL) than their non-OSA counterparts. Leptin showed a correlation with OSA severity markers. OSA was associated with greater cardiac damage in AMI patients. Our findings underscore that adipokines alone are not sufficient to discriminate the risk of AMI in the presence of OSA. Further research is necessary to determine the potential mechanisms contributing to exacerbated cardiac damage in patients with both conditions.

## 1. Introduction

Acute coronary syndrome (ACS) represents a significant global health burden, ranking among the foremost causes of morbidity and mortality worldwide. Within the spectrum of ACS, acute myocardial infarction (AMI) stands out as a critical event resulting from the interplay of coronary and acute vascular thrombosis. Despite clinical efforts, AMI and stroke continue to account for approximately 80% of all cardiovascular-related deaths [1]. The early identification of patients at risk holds promise for timely intervention and improved clinical outcomes [2].

Compelling evidence demonstrates a strong association between AMI and obstructive sleep apnea (OSA) [3]. About 65% of patients presenting with cardiovascular complications are concurrently diagnosed with OSA [4]. Likewise, OSA increases the risk of cardiovascular events 1.5-fold in the general population and up to 2.6-fold in high-risk populations [5]. OSA is characterized by recurrent upper airway obstruction and intermittent hypoxia, leading to chronic sleep fragmentation and impaired sleep quality [6]. Within the OSA classification, several subtypes exist, driven by multiple risk factors, including anatomical, physiological, inflammatory, and obesity-related factors [7]. Obesity results in the excessive accumulation of adipose tissue within the upper airway walls, exacerbating ventilatory collapse during sleep [8]. Furthermore, adipose tissue functions as a highly active endocrine organ that secretes biologically active signaling molecules known as adipokines [9]. Adipokines are key mediators in energy regulation, fat accumulation, and inflammation, which are relevant processes in the pathogenesis of cardiometabolic disorders [9]. 

Both obesity and OSA are directly associated with elevated levels of inflammatory markers, including C-reactive protein (CRP) and interleukin (IL)-6 [10], substantially increasing the risk of cardiovascular disease. In parallel, obesity-related adipose tissue actively participates in the synthesis of inflammatory factors such as leptin and resistin, while concurrently inhibiting the secretion of anti-inflammatory mediators like adiponectin [11]. A recent meta-analysis, encompassing pediatric and adult populations, identified a significant association between increased adipokines and inflammation markers, further underscoring their contribution to the pathophysiology of OSA [12]. Adipokines, particularly leptin and adiponectin, act on various molecular targets in response to OSA, mediating systemic metabolic changes in response to physiological perturbations and sleep disturbances [13].

Moreover, OSA has been linked to components of metabolic syndrome, including hypertension, insulin resistance, and dyslipidemia [14]. However, the deleterious impact of OSA extends beyond metabolic dysfunction, significantly elevating the risk of stroke, AMI, heart failure, arrhythmias, sudden cardiac death, and all-cause mortality [15].

Given the high prevalence of impaired metabolic health worldwide, this research aims to investigate the potential association between adipokines, OSA, and cardiovascular health. By analyzing the circulating adipokine profile, this study seeks to deepen our understanding of the intricate interplay among OSA, adipokines, and the pathogenesis of AMI. Specifically, we aim to determine serum adipokine levels in AMI patients with and without OSA, as well as in non-AMI individuals with and without OSA.

## 2. Results

### 2.1. Clinical Features

A total of 55 participants were included in this study, consisting of 30 patients with AMI (83% men, median age 56 (30–61) years) and 25 controls (84% men, age 51 (45–57) years). Both groups exhibited a high prevalence of comorbidities, although a higher incidence of dyslipidemia (20% vs. 0; *p* = 0.026) and smoking (60% vs. 4%; *p* < 0.001) was observed among the AMI group. Additionally, glucose levels were higher in the AMI group, while lipid levels showed no differences. Both the AMI and control groups exhibited poor sleep quality as assessed by the PSQI and sleepiness assessment. Notably, the AMI group demonstrated a higher risk of suffering from OSA based on the Berlin test. The key clinical characteristics are summarized in Table 1.

Furthermore, we conducted a subgroup analysis within the AMI group based on the presence (*n* = 15) or absence (*n* = 15) of OSA (Table 2). No significant differences were observed in the frequency of comorbidities, metabolic markers, pharmacological therapy, or body composition between these groups. However, patients with AMI and OSA exhibited elevated levels of CK (1430, 301–5432 vs. 377, 125–2194 U/L; *p* = 0.036), CK-MB (64, 15–300 vs. 9, 4–148 ng/mL; *p* = 0.044), and cTnT (2298, 382–17854 vs. 356, 142–1021 pg/mL; *p* = 0.041).

### 2.2. Sleep Quality

Our assessment of sleep quality in all groups, using clinical questionnaires, revealed that both the AMI and control groups displayed poor sleep quality according to the PSQI. These findings were consistent regardless of the presence or absence of OSA. The data are summarized in Table 3.

### 2.3. Home Respiratory Polygraphy 

We examined the differences in respiratory patterns through HRP. Among individuals with OSA, the AHI was 20.9 (15.4–31) in AMI patients and 28.8 (9.2–47.9) in the controls; however, these differences were not statistically significant. Notably, the controls with OSA experienced a higher number of obstructive events (90, 59–178 vs. 57, 30–90; *p* = 0.031), while central apneas were more frequent in AMI patients with OSA (1, 0–3 vs. 0, 0–0; *p* = 0.016). The data are summarized in Table 4.

### 2.4. Serum Levels of Inflammatory Mediators

Resistin levels were found to be significantly higher in the AMI groups compared to the Control w/o OSA group: AMI w/o OSA vs. Control w/o OSA (7.4 ± 3.4 vs. 3.69 ± 1.2; *p* = 0.030) and AMI w OSA vs. Control w/o OSA (7.15 ± 4.2 vs. 3.69 ± 1.2; *p* = 0.039). 

Similarly, IL-6 levels were elevated in the AMI groups compared to the Control w/o OSA group: AMI w/o OSA vs. Control w/o OSA (8.80 ± 11.1 vs. 1.35 ± 1.17; *p* = 0.003) and AMI w/OSA vs. Control w/o OSA (8.94 ± 9.1 vs. 1.35 ± 1.17; *p* = 0.003).

**Figure 1:**
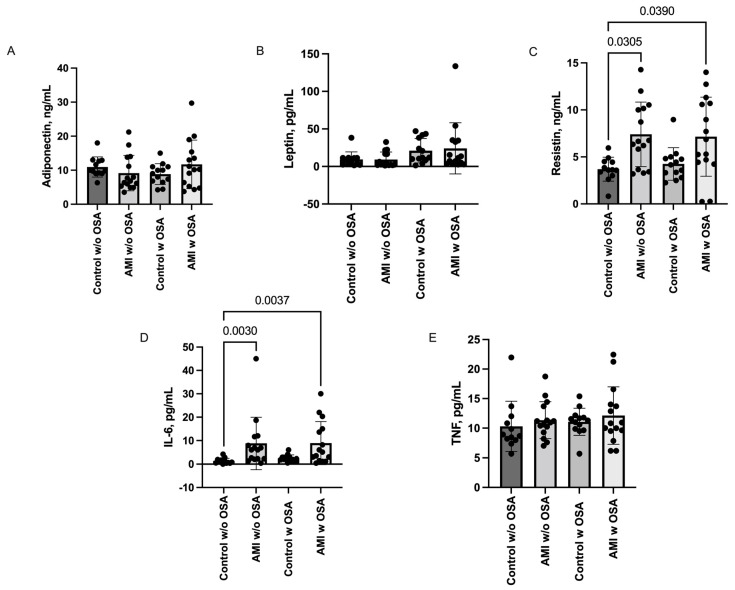
Serum levels of adipokines (**A**) adiponectin, (**B**) leptin, (**C**) resistin, (**D**) IL-6, and (**E**) TNF-α in all groups. Data are presented as means with SDs. Differences between groups were analyzed via Kruskal-Wallis and Dunn’s multiple comparison test. *p* values < 0.05 were considered to represent statistical differences. Interleukin-6 (IL-6), Tumor Necrosis Factor α (TNF).

No significant differences were observed between groups for the adiponectin, leptin, and TNF-α levels. Graphical representations of these data can be seen in Figure 1, and detailed values are provided in the Supplementary Table S1.

We also investigated inflammatory markers related to cardiac damage and observed an increase in ET-1 levels in AMI patients, regardless of the presence of OSA: Control w/o OSA vs. AMI w/o OSA (1.83 ± 0.35 vs. 3.31 ± 1.42; *p* = 0.0005) and Control w/o OSA vs. AMI w OSA (3.31 ± 1.42 vs. 3.22 ± 2.10; *p* = 0.026). Additionally, subjects w/o AMI and w/o OSA exhibited lower levels of PAI-1 and SAA compared to the Control w OSA group.

**Figure 2:**
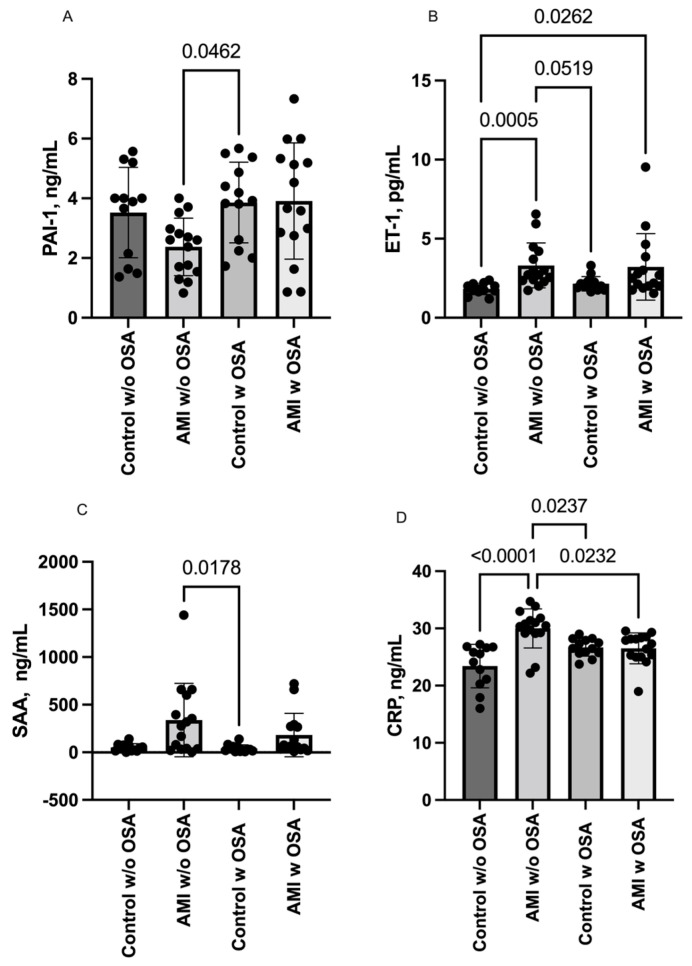
Serum levels of inflammatory markers (**A**) PAI-1, (**B**) ET-1, (**C**) SAA, and (**D**) CRP in all the groups. Data are presented as means with SDs. Differences between groups were analyzed via Kruskal-Wallis and Dunn’s multiple comparison test. *p* values < 0.05 were considered to represent statistically significant differences. Plasminogen activator inhibitor 1 (PAI-1); Endothelin 1 (ET-1); Serum Amyloid A (SAA), and C-reactive protein (CRP).

In contrast, CRP levels increased in AMI patients w/o OSA: AMI w/o OSA vs. Control w/o OSA (30.0 ± 3.41 vs. 23.42 ± 3.79; *p* < 0.0001). Interestingly, CRP levels in AMI patients w/o OSA were higher than in groups w OSA: AMI w/o OSA vs. Control w OSA (30.0 ± 3.41 vs. 26.68 ± 1.54; *p* = 0.024) and AMI w/o OSA vs. AMI w OSA (30.0 ± 3.41 vs. 26.5 ± 2.69; *p* = 0.023). These results are graphically represented in Figure 2, and detailed values are provided in the Supplementary Table S1. 

### 2.5. Correlation of Adipokine Levels with Biomarkers for Clinical Use and Risk Scales

To explore the possible associations between adipokine levels, cardiac risk markers, and OSA severity markers, we conducted correlations analyses.

#### 2.5.1. AMI Patients

**Figure 3:**
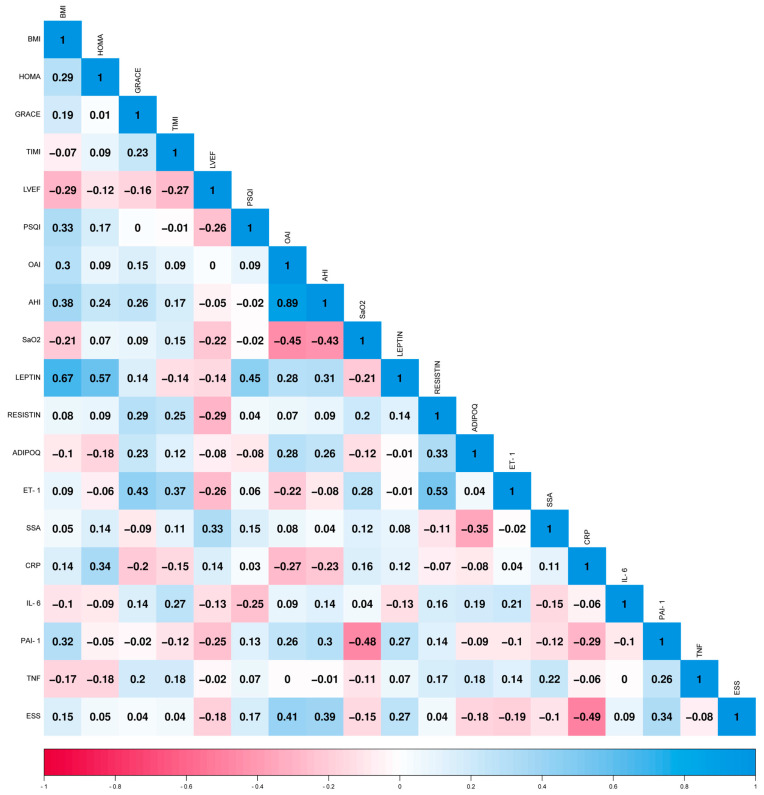
Correlation matrix of AMI patients, with the serum levels of inflammatory markers and the main indices. Spearman’s correlation coefficients are presented. The heatmap was plotted using https://www.bioinformatics.com.cn/en (accessed on 19 September 2023), a free online platform for data analysis and visualization. Body mass index (BMI); Homeostatic Model Assessment (HOMA); The Global Registry of Acute Coronary Events (GRACE); thrombolysis in myocardial infarction (TIMI); Left Ventricular Ejection Fraction (LVEF); Pittsburgh Sleep Quality Index (PSQI); Obstructive Apnea Index (OAI); Apnea Hypopnea Index (AHI); Saturation of Oxygen (SaO_2_); Endothelin-1 (ET-1); Serum amyloid A (SAA); C-reactive protein (CRP); Interleukin-6 (IL-6); Plasminogen Activator Inhibitor 1 (PAI); Tumor Necrosis Factor α (TNF); Epworth Sleepiness Score (ESS); Creatine Kinase (CK); Creatin Kinase isoenzyme MB (CK-MB); and Cardiac troponin T (TnT).

Among all the AMI patients (*n* = 30), we observed correlations between leptin levels and BMI (0.67), HOMA (0.57), and PSQI (0.45). ET-1 levels correlated with the GRACE (0.43) and TIMI (0.37) risk scores. Conversely, CRP negatively correlated with ESS (−0.49). These correlations are graphically represented in Figure 3.

#### 2.5.2. Control Subjects

**Figure 4:**
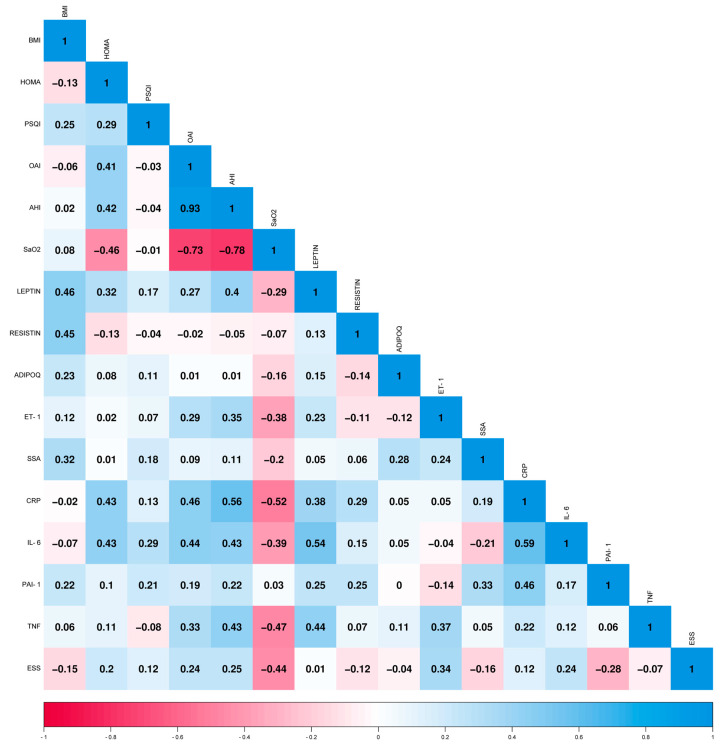
Correlation matrix of control group, with the serum levels of inflammatory markers and the main indices. Spearman’s correlation coefficients are presented. The heatmap was plotted using https://www.bioinformatics.com.cn/en (accessed on 19 September 2023), a free online platform for data analysis and visualization. Body mass index (BMI); Homeostatic Model Assessment (HOMA); Pittsburgh Sleep Quality Index (PSQI); Obstructive Apnea Index (OAI); Apnea Hypopnea Index (AHI); Saturation of Oxygen (SaO_2_); Endothelin-1 (ET-1); Serum amyloid A (SAA); C-reactive protein (CRP); Interleukin-6 (IL-6); Plasminogen Activator Inhibitor 1 (PAI); Tumor Necrosis Factor α (TNF); Epworth Sleepiness Score (ESS).

In the control group (*n* = 25), we found associations between adipokines and OSA severity markers. Leptin correlated with BMI (0.46). CRP correlated with HOMA (0.43), OAI (0.46), and AHI (0.56). IL-6 correlated with HOMA (0.43), OAI (0.44), and AHI (0.43). Resistin correlated with BMI (0.45), while TNF-α correlated with AHI (0.43). These correlations are presented in Figure 4.

We conducted a final set of analyses to explore the influence of sleep components on the adipokine levels. In the AMI patient group, we found that TNF-α levels positively correlated with sleep efficiency (0.43). Conversely, among subjects w/o AMI, PAI-1 correlated with sleep quality (0.40), adiponectin with sleep efficiency (0.47), SAA with sleep efficiency (0.44), ET-1 with sleep disturbance (0.40), and IL-6 with sleep disturbance (0.42).

## 3. Discussion

In this study, we aimed to investigate the association between circulating adipokine and inflammatory marker levels and the presence of OSA in patients experiencing their first AMI. Our findings provide valuable insights into the complex interplay between sleep disorders, cardiovascular risk, and adipokine levels.

We observed that adipokine levels alone are not a definitive indicator of OSA prevalence in AMI. However, cardiac risk markers are higher in AMI patients with OSA, and adipokine levels correlate with OSA severity markers. Additionally, we identified notable deficiencies in sleep patterns among all study participants, with a self-reported average sleep duration of 5.4 h per night. This sleep duration falls below the recommended threshold of 6 h, and previous research has associated such short sleep durations with a 38% increase in the incidence of obesity in adults [16]. We assessed sleep quality using the PSQI, which indicated substantial sleep problems among our study participants, as reflected by an average PSQI score of 9.28 [17].

Sleep disorders are recognized as major determinants of sleep quality, and their impact on overall health is a growing concern across all age groups. Sleep disorders have been identified as risk factors for conditions such as metabolic syndrome, coronary heart disease, and systemic hypertension [18]. Notably, OSA has established links with cardiovascular disorders, with high prevalence rates observed in patients with various cardiovascular conditions, including hypertension, AMI, arrhythmias, and coronary artery disease [19]. Our study underscored the need for the early diagnosis of OSA to mitigate cardiovascular risk.

Strikingly, our control group exhibited a high prevalence of OSA (52%), potentially attributable to a high rate of overweight individuals within the group. Moreover, it is likely that undiagnosed metabolic alterations were present due to elevated blood lipid levels. Our investigation revealed a high prevalence of OSA among patients experiencing their first AMI, similar to that found in non-infarcted subjects. This finding raises awareness about the elevated cardiovascular risk in our adult population, attributed to the coexistence of metabolic comorbidities and the substantial prevalence of OSA. Additionally, we noted the occurrence of early-onset AMI, likely influenced by the combination of high smoking rates and current lifestyle patterns (as indicated in Table 1, Table 2 and Table 3). These circumstances emphasize the importance of addressing OSA clinically, as it has not yet received the prioritized attention it deserves in the prevention and treatment of coronary artery disease. In addition, higher levels of abdominal fat, increased incidence of insulin resistance, and alterations in lipid metabolism were observed in these subjects. Almost all of our population was overweight, which is a relevant situation because overweight and obesity are considered the main risk factors for OSA [20]. Adipose tissue plays an important role in the pathogenesis of cardiovascular disease through the production of adipokines. In the presence of obesity, adipokines alter their protective profile and generate harmful effects on peripheral tissues and the cardiovascular system [21]. Therefore, it has been proposed that OSA may modulate adipokine production in adipose tissue [22]. Obesity stands as a strong risk factor for cardiovascular disease, inducing airway lumen narrowing, oxidative stress, endothelial damage, platelet activation, and elevated inflammatory mediators [7].

The role of adipokines in OSA remains a subject of debate, with clinical studies yielding conflicting results and even inconclusive meta-analyses. Leptin, one of the most extensively studied adipokines, is often linked to OSA. While leptin levels tend to increase with OSA severity, this association is likely confounded by obesity rather than OSA itself [10]. In our study, serum leptin levels did not significantly differ between groups, but they were correlated with BMI independently of OSA and AMI. Moreover, leptin levels correlated with PSQI, OAI, SaO_2_, and sleep quality in subjects with and without OSA. These findings suggest that leptin may play a role in sleep quality and OSA.

Adiponectin, another frequently studied adipokine, is believed to decrease with OSA severity [10], although obesity may also contribute to this trend. In our study, we did not observe significant changes in circulating adiponectin levels. However, among subjects without OSA, we identified a correlation between adiponectin levels and sleep efficiency.

Resistin, another adipokine of interest, is increased in OSA patients and its levels have been positively correlated with disease severity [10]. Resistin production is influenced by inflammatory factors such as IL-6 and TNF-α [23]. In our study, resistin levels increased in AMI patients, regardless of the presence of OSA, along with IL-6 levels. Moreover, IL-6 has been shown to increase substantially after AMI, in association with poor short-term outcomes [24]. The rise in IL-6 levels may stem from various sources, including vascular cells, endothelial cells, adipocytes, activated immune cells, and even cardiomyocytes, suggesting that IL-6 and resistin may jointly contribute to AMI progression [25]. These findings suggest that IL-6 and resistin participate jointly in the progression of AMI, by subserving the development of atherosclerosis and the inflammatory process, positioning them as potential prognostic biomarkers [26].

The role of adipokines in infarction has not been fully elucidated, although a deleterious cardiovascular effect has been observed in animal models [27]. A study focused on measuring adipokines in AMI patients found that their levels depend on the severity of the infarct, with a direct relationship with resistin and an inverse relationship with adiponectin. Furthermore, it was found that resistin had prognostic significance for recurrent ACS, and that its levels were related to the extent of inflammation [28]. Resistin has also been associated with increased inflammation by inducing CRP secretion [10]. We found that resistin levels are increased in AMI, in association with elevated levels of non-adipose tissue molecules such as ET-1 and CRP. These observations are in parallel with previous evidence. Furthermore, leptin levels correlated positively with BMI, HOMA, and PSQI in AMI patients. In those with AMI and OSA, leptin also exhibited a correlation with SaO_2_ and negative associations with sleep duration, highlighting the complex interplay between OSA and AMI.

Inflammation and OSA are interconnected and jointly participate in the progression of cardiovascular disease. Elevated levels of IL-6 and CRP have been observed in OSA patients, indicating that inflammatory markers are present from the early stages of the disease [29]. These markers may participate in endothelial dysfunction through increased ET-1 production [30]. In our study, OSA was associated with elevated levels of myocardial lysis markers (CK, CK-MB, and cTnT) and ET-1. These increases are important as they suggest that OSA carries an elevated risk of myocardial damage in cardiovascular disease. Nevertheless, it is worth noting that the relationship between OSA and myocardial markers is complex, with contradictory findings in the literature. Some studies have reported lower troponin levels in OSA patients presenting with AMI, suggesting a cardioprotective effect [31]. Additionally, meta-analyses have failed to identify significant differences in troponin, CK, and CK-MB levels between cardiovascular disease patients with and without OSA [32]. Our results contribute to this ongoing controversy since CRP levels were higher in AMI patients without OSA compared to those with OSA. In addition, CRP levels correlated with ESS in AMI patients w/OSA.

When relating quality of sleep with adipokines, we found that quality of sleep correlates with some molecules such adiponectin, SAA, TNF-α, and IL-6. A broad spectrum of disorders characterized by low-grade inflammatory upregulation has been identified, including cardiovascular and metabolic disorders. However, the precise initiating trigger for this inflammatory upregulation remains elusive. Notably, a substantial body of evidence has accumulated, suggesting that sleep deficiency may serve as a potent risk factor in this context [33]. 

Our study is limited in several ways. Firstly, the small sample size and the recruitment of patients only in Mexico City restrict the generalizability of our findings. Secondly, HRP was performed one month after the occurrence of AMI, limiting our ability to establish simultaneous associations. Thirdly, an unusually high frequency of undiagnosed OSA was observed among the control subjects, which could potentially confound comparisons with AMI patients. Lastly, we did not conduct serial measurements of adipokines and other inflammatory mediators, which could have provided a more comprehensive understanding of their dynamics over time. Despite these methodological shortcomings, our results support that patients with OSA have greater cardiac damage during an AMI, and the analysis of adipokines opens new avenues of analysis as they stand out as indicators of the severity of OSA and cardiac injury.

## 4. Material and Methods

### 4.1. Study Participants

We conducted a prospective case–control study of consecutive patients. Between January 2021 and January 2022, we recruited adult patients with AMI from the coronary care unit of the Instituto Nacional de Cardiología Ignacio Chávez in Mexico City, a national reference center devoted to cardiovascular diseases and allied conditions. To be included, patients had to be fully conscious, hemodynamically stable, experiencing their first AMI, and within 24 h of symptom onset. 

Exclusion criteria: (1) pregnant patients; (2) chronic inflammatory or autoimmune diseases; (3) chronic kidney disease; (4) stroke; (5) neoplasia or active infection; (6) patients unable to undergo respiratory polygraphy during follow-up; (7) respiratory polygraphy duration of less than 240 min; (8) patients with a history of ischemic vascular disease.

Control Group: unrelated individuals without pre-existing cardiac conditions, matched for age and sex, were included for comparative analysis.

#### Ethical Approval

The study protocol was approved by the local ethics committee (No. 21-1208) and participants provided informed consent. All procedures were performed following the principles of the 2013 Declaration of Helsinki and local regulations.

Diagnostic and treatment decisions were made at the discretion of the treating physician, including medical procedures and hospital discharge.

### 4.2. AMI Diagnosis

Patients with a diagnosis of AMI were classified as either having ST-segment elevation myocardial infarction (STEMI) or non-ST-segment elevation myocardial infarction (NSTEMI) based on clinical characteristics, electrocardiographic changes, and biochemical markers of cardiac necrosis (specifically, creatinine kinase-MB (CK-MB) or troponin T (Tn), according to the standard definitions set by the American College of Cardiology and the European Society of Cardiology [34,35]).

#### 4.2.1. Clinical Classification of AMI

AMI was further categorized based on characteristics and Left Ventricular Ejection Fraction (LVEF), which is a central measure of left ventricular systolic function [36]. LVEF was classified as follows:

Hyperdynamic = LVEF greater than 70%.

Normal = LVEF 50% to 70% (midpoint 60%).

Mild dysfunction = LVEF 40% to 49% (midpoint 45%).

Moderate dysfunction = LVEF 30% to 39% (midpoint 35%).

Severe dysfunction = LVEF less than 30%.

#### 4.2.2. Risk Scale for AMI

We calculated the GRACE score (Global Registry of Acute Coronary Events) for all patients, considering age, heart rate, systolic blood pressure, plasma creatinine concentration, Killip–Kimball class at admission, cardiac arrest at admission, ST-segment changes, and elevated myocardial necrosis markers. Patients were classified into three risk groups based on their GRACE scores: I—low risk (≤109 points), II—intermediate risk (110–139 points), and III—high risk (≥140 points) [37]. The Killip–Kimball (K-K) classification was also applied to estimate acute heart failure severity, stratifying patients into four groups [38].

### 4.3. Laboratory Procedures

Once the patients consented to participating in the study and before performing the cardiac catheterization, a total of 6 mL of peripheral venous blood was collected using tubes containing coagulation-activating gel. Blood samples were centrifuged, and the resultant sera were aliquoted and stored at −80 °C until use. Sera were thawed under standard conditions, and the levels of inflammatory markers were assessed using enzyme-linked immunosorbent assays, following the manufacturer’s recommended protocols. Adipokines: adiponectin (Millipore, Darmstadt, Germany; detection range 1.5–100 ng/mL), leptin (Millipore; 1.64–400 pg/mL), resistin (R&D Systems, Minneapolis, MN, USA; 0.2–10 ng/mL), IL-6 (R&D Systems; 3.1–300 pg/mL), and tumoral necrosis factor (TNF-α; Invitrogen, Vienna, Austria; 0.5–32 pg/mL). Cardiac Markers: plasminogen activator inhibitor-1 (Serpin E1/PAI-1; R&D Systems; 0.3–20 ng/mL), endothelin-1 (ET-1; R&D Systems; 0.4–25 pg/mL), serum amyloid A (SAA; Invitrogen, Frederick, MD, USA; 9.4–600 ng/mL), and CRP (RayBio, Norcross, GA, USA; 34.29–25,000 pg/mL). 

Additionally, the levels of CK (40–129 U/L), CK-MB (0.3–4.88 ng/mL), pro-brain natriuretic peptide (Pro-BNP; 5–162.4 pg/mL), and cardiac troponin T (cTnT; 3–14 pg/mL) were measured using the Roche Cobas c701 automatic analyzer (Roche Diagnostics, Mannheim, Germany).

### 4.4. Sleep Quality Assessment

The assessment of sleep quality was conducted during the hospital stay in the coronary care unit. The following questionnaires, translated into Spanish and already validated, were used:

Pittsburgh Sleep Quality Index (PSQI) [39]: This a self-report assessment tool that evaluates sleep quality over a one-month period. This score comprises seven components (sleep quality, sleep latency, sleep duration, sleep efficiency, sleep disturbance, sleep medication, and daily dysfunction). Each component is scored on a scale from 0 to 3, resulting in a total score ranging from 0 to 21. A total PSQI score greater than 5 has been accepted as indicative of poor sleep quality [40]. 

Berlin Questionnaire [41]: This questionnaire consists of 10 questions categorized into snoring behavior, daytime sleepiness or fatigue, and the presence of obesity or hypertension. Patients exhibiting persistent and frequent symptoms (>4 times per week) in at least two of the three categories are classified as being at high risk of developing sleep apnea [42].

Epworth Sleepiness Scale (ESS) [43]: A validated questionnaire used in research and clinical practice, the ESS asks subjects to rate their likelihood of falling asleep in eight different scenarios. Higher scores indicate a greater propensity to fall asleep, with a normal score ranging between 0 and 10. Scores above 10 warrant further medical assessment [44]. 

We established the sleep periods as short sleep (<6 h/night), normal sleep (7–8 h/night), and long sleep (>9 h/night).

### 4.5. Home Respiratory Polygraphy

All study participants underwent home respiratory polygraphy (HRP) using Alice NightOne (Philips Respironics, Weinmann, Germany) equipment, based on the analysis of respiratory events (apneas/hypopneas) during overnight monitoring. For patients with AMI, HRP was performed 30 days after hospital discharge.

Apnea was defined as a >90% reduction in the respiratory flow signal for at least 10 s, while hypopnea was defined as a drop in the respiratory flow signal of >30% for at least 10 s accompanied by oxygen desaturation of >3% [45]. The evaluated parameters included the apnea/hypopnea index (AHI), defined as the total number of respiratory events (apnea/hypopnea) divided by the overall study time in hours; obstructive apnea index (OAI), representing the number of obstructive apneas per hour of sleep; oxygen desaturation index (ODI), indicating the number of oxygen saturation drops of >4% per hour of study; and the lowest oxygen saturation during sleep (SaO_2_). Polygraphy parameters were acquired and manually processed by a single operator (JE-A).

### 4.6. Diagnosis of Obstructive Sleep Apnea

The diagnosis of OSA was established in accordance with the International Consensus Document on Obstructive Sleep Apnea. Specifically, it was based on either of the following criteria: (1) the presence of an AHI of ≥15 events/hour, primarily obstructive; or (2) AHI ≥ 5, accompanied by one or more of the following factors: excessive daytime sleepiness, non-restorative sleep, excessive tiredness, and/or impaired sleep-related quality of life, with no explanation from other causes [46].

### 4.7. Body Composition Analysis

Upon conclusion of the HRP recording, a body composition analysis was performed using the Avis 333 Plus Segmental Body Composition Analyzer (Jawon Medical, Seoul, Republic of Korea). This assessment included body weight, body fat percentage, body mass index (BMI), lean body mass, and waist/hip ratio (WHR).

### 4.8. Statistical Analysis

Data distribution was evaluated using the D’Agostino–Pearson test, revealing a non-normal distribution. Consequently, qualitative variables are presented as percentages, while quantitative variables are described using the median and interquartile range (mean and standard deviation for inflammatory markers). Differences between groups were analyzed using Fisher’s exact test, the Mann–Whitney test, and the Kruskal–Wallis test with Dunn’s multiple comparison test as appropriate. Correlation analyses utilized Spearman’s rho coefficients.

All analyses were two-tailed, and a *p* value < 0.05 was set for statistical significance. Statistical analyses and graph creation were performed using GraphPad Prism v9.4 (GraphPad Inc., La Jolla, CA, USA). The heatmap was created through an online platform for data analysis and visualization (https://bioinformatics.com.cn/en (accessed on 19 September 2023)).

## 5. Conclusions

Our study reveals that adipokines alone are not sufficient to discriminate the risk of an ACS in the presence of OSA. Among the adipokines studied, leptin emerges as being most closely related to the severity of apnea, while the levels of IL-6, ET-1, and resistin are elevated during AMI regardless of OSA’s presence. Finally, our findings highlight the correlation between adipokine levels and poor sleep quality.

## Figures and Tables

**Table 1:** Main clinical characteristics of AMI patients compared with the control group.

Clinical Features			
	AMI *n* = 30	Control *n* = 25	*p*
Men, n (%)	25 (83)	21 (84)	>0.999
Age, years Median (IQR)	56 (30–61)	51 (45–57)	0.069
Diabetes, n (%)	11 (36.7)	4 (16)	0.129
Hypertension, n (%)	13 (43.3)	7 (28)	0.273
Dyslipidemia, n (%)	6 (20)	0 (0)	0.026
Smoking, n (%)	18 (60)	1 (4)	<0.001
Overweight, n (%)	8 (26.7)	19 (76)	<0.001
Obese, n (%)	13 (43.3)	6 (24)	0.163
WHR Median (IQR)	0.97 (0.9–1.0)	1.01 (0.9–1.0)	0.170
Glucose (mmol/L) Median (IQR)	6.41 (5.8–8.3)	5.55 (5.0–6.0)	<0.001
HOMA index Median (IQR)	4.1 (2.9–10)	3.7 (2.4–11)	0.096
Triglycerides (mg/dL) Median (IQR)	177 (120.8–215.5)	165 (130–293)	0.824
Total cholesterol (mg/dL) Median (IQR)	138 (124.5–175)	194 (157.5–216.5)	<0.001
HDL (mg/dL) Median (IQR)	32.95 (28.4–38)	41.3 (37.2–48.9)	<0.001
LDL (mg/dL) Median (IQR)	86.8 (68.8–106.5)	118 (95.2–136.5)	0.006
**Sleep Quality**			
Sleep quantity, (hours) Median (IQR)	6 (4.7–7)	5 (4.7–8)	0.241
Global PSQI score Median (IQR)	10 (5.7–13)	10 (6–11.5)	0.707
ESS Median (IQR)	9 (5.7–14.2)	10 (6–11.5)	0.683
Berlin Questionnaire, n (%)	15 (50)	7 (28)	0.016
AHI Median (IQR)	13.5 (6.7–21)	17.3 (6.7–28.8)	0.635

Qualitative variables are presented as percentages and quantitative variables are presented as medians and interquartile ranges. Differences between groups were analyzed via Fisher’s exact test and Mann–Whitney test, respectively. *p* values < 0.05 were considered to represent statistically significant differences. Waist-to-hip ratio (WHR), Homeostasis Model Assessment (HOMA), high-density lipoprotein (HDL), low-density lipoprotein (LDL), Pittsburgh Sleep Quality Index (PSQI), Epworth Sleepiness Scale (ESS), Apnea Hypopnea Index (AHI).

**Table 2:** Main clinical characteristics of AMI patients without OSA (AMI w/o OSA) and with OSA (AMI w OSA).

Clinical Features			
	AMI w OSA *n* = 15	AMI w/o OSA *n* = 15	*p*
Sex, men (%)	13 (86.7)	12 (80)	>0.999
Age (years) Median (IQR)	56 (53–62)	54 (45 -58)	0.183
Diabetes, n (%)	8 (61.5)	3 (20)	0.051
Hypertension, n (%)	8 (53.3)	5 (33.3)	0.462
Dyslipidemia, n (%)	3 (20)	3 (20)	>0.999
Smoking, n (%)	8 (53.3)	10 (66.7)	0.710
Overweight, n (%)	3 (20)	5 (33.3)	0.682
Obese, n (%)	8 (53.3)	5 (33.3)	0.462
WHR Median (IQR)	0.98 (0.96–1.0)	0.96 (0.93–0.99)	0.227
Glucose (mmol/L) Median (IQR)	7.4 (5.9–8.5)	6.2 (5.9–7.0)	0.361
HOMA index Median (IQR)	8.8 (3–11)	4 (2.8–7)	0.244
Triglycerides (mg/dL) Median (IQR)	179 (114–215)	176 (122–217)	0.713
Total cholesterol (mg/dL) Median (IQR)	134 (123–176)	150 (125–179)	0.418
HDL (mg/dL) Median (IQR)	33.4 (28.3–37.6)	32 (28.4–39.1)	0.829
LDL (mg/dL) Median (IQR)	86.6 (65.2–106)	91.9 (76.3–123)	0.486
CK (U/L) Median (IQR)	1430 (301–5432)	377 (125–2194)	0.036
CK-MB (ng/mL) median (IQR)	64.6 (15.1–300)	9.7 (4.3–148)	0.044
Pro-BNP (pg/mL) Median (IQR)	247 (115–854)	351 (170–768)	0.653
cTnT (pg/mL) Median (IQR)	2298 (382–17854)	356 (142–1021)	0.041
Type of heart attack (STEMI), n (%)	13 (86.7)	9 (60)	0.215
GRACE Median (IQR)	101 (95–115)	95 (77–109)	0.221
% LVEF Median (IQR)	54 (38–57)	54 (45–56)	0.628
K-K [I], n (%)	12 (80)	13 (86.7)	>0.999
**Basic therapy**			
Lipid-lowering agents, n (%)	2 (13.3)	0 (0)	0.483
Insulin, n (%)	1 (6.7)	1 (6.7)	>0.999
Metformin, n (%)	4 (26.7)	3 (20)	>0.999
Antihypertensives, n (%)	6 (40)	3 (20)	0.427
ASA, n (%)	0 (0)	2 (13.3)	0.483
**Emergency therapy**			
DAPT, n (%)	9 (45)	9 (45)	>0.999
Beta-blockers, n (%)	4 (26.7)	3 (20)	>0.009
ACEi/ARA II, n (%)	5 (33.3)	4 (26.7)	>0.999
Anticoagulant, n (%)	6 (40)	5 (33.3)	>0.999
Statin, n (%)	11 (73.3)	10 (66.7)	>0.999

Qualitative variables are presented as percentages and quantitative variables are presented as medians and interquartile ranges. Differences between groups were analyzed via Fisher’s exact test and Mann–Whitney test, respectively. *p* values < 0.05 were considered to represent statistically significant differences. Waist-to-hip ratio (WHR), Homeostasis Model Assessment (HOMA), high-density lipoprotein (HDL), low-density lipoprotein (LDL), creatine kinase (CK), creatin kinase isoenzyme MB (CK-MB), brain natriuretic peptide (BNP), cardiac troponin T (cTnT), Global Registry of Acute Coronary Events (GRACE), Left Ventricular Ejection Fraction (LVEF), Killip–Kimball (K-K), Acetylsalicylic acid (ASA), dual antiplatelet therapy (DAPT), angiotensin-converting enzyme inhibitors (ACEi), Angiotensin II receptor antagonist (ARA II).

**Table 3:** Sleep characteristics.

Sleep Quality						
	AMI w OSA *n* = 15	Control w OSA *n* = 13	*p*	AMI w/o OSA *n* = 15	Control w/o OSA *n* = 12	*p*
Sleep quality Median (IQR)	1 (1–2)	1 (1–2)	0.451	1 (1–2)	1 (1–1.75)	0.951
Sleep latency Median (IQR)	1 (0–2)	1 (0–2.5)	0.631	2 (0–3)	1.5 (0.2–2)	0.299
Sleep duration Median (IQR)	2 (0–2)	2 (2–3)	0.083	2 (1–3)	2 (2–3)	0.451
Sleep efficiency Median (IQR)	2 (0–3)	1 (0–2)	0.258	2 (0–3)	1 (0–2)	0.568
Sleep disturbance Median (IQR)	2 (1–2)	2 (1–2)	0.258	2 (1–2)	1.5 (1–2)	0.625
Sleep medication Median (IQR)	0 (0–0)	0 (0–0)	0.484	0 (0–0)	0 (0–0)	0.487
Daily dysfunction Median (IQR)	1 (0–2)	2 (0–3)	0.752	2 (0–3)	1 (1–2.7)	0.971
Global PSQI score Median (IQR)	10 (4–13)	9 (6–12)	0.936	10 (7–13)	10 (2.2–10.5)	0.602
ESS Median (IQR)	13 (6–17)	12 (6.5–14)	0.516	7 (4–11)	9 (4–12.5)	0.636
Berlin Questionnaire, n (%)	9 (64.3)	4 (30.8)	0.128	6 (40)	4 (33.3)	>0.999

Data are presented as medians and interquartile ranges, and percentages in the case of Berlin Questionnaire. Differences between groups were analyzed via Kruskal–Wallis test and Chi-square test, respectively; *p* values < 0.05 were considered to represent statistically significant differences. No significant difference were observed between groups for the PSQI score and Berlin Questionnaire. OSA patients had elevated Epworth Sleepiness Scores in comparison with w/o OSA patients, which highlights poor quality of sleep in general population. Pittsburgh Sleep Quality Index (PSQI); Epworth Sleepiness Scale (ESS).

**Table 4:** Respiratory polygraphy.

	AMI w OSA *n* = 15	Control w OSA *n* = 13	*p*	AMI w/o OSA *n* = 15	Control w/o OSA *n* = 12	*p*
AHI, events per h Median (IQR)	20.9 (15.4–31)	28.8 (19.2–47.9)	0.115	6.9 (4.5–11.6)	6.7 (4.3–8.0)	0.493
Total number of obstructive events Median (IQR)	57 (30–90)	90 (59–178)	0.032	14 (5–31)	5.5 (4–17.7)	0.076
Number of central apneas Median (IQR)	1 (0–3)	0 (0–0)	0.016	0 (0–0)	0 (0–0.7)	0.932
Number of hypopneas Median (IQR)	74 (53–107)	65 (54–103.5)	0.973	33 (29–51)	30.5 (14.7–42.2)	0.304
Total respiratory events Median (IQR)	246 (118–188)	185 (140–262)	0.204	48 (37–89)	39 (25–55)	0.203
OAI Median (IQR)	8.6 (4.8–17.5)	18.2 (8.8–30.5)	0.047	1.9 (0.7–3.9)	0.9 (0.5–2.8)	0.231
CAI Median (IQR)	0.1 (0–0.4)	0 (0–0)	0.014	0 (0–0)	0 (0–0.07)	0.932
Lowest SpO_2_ during sleep Median (IQR)	73 (68–77)	69 (65–74.5)	0.221	80 (75–84)	80 (76.8–81.7)	0.691
ODI > 4%, per h Median (IQR)	45 (25.6–48.5)	43.6 (28.2–58)	0.488	18.1 (16.3–26.5)	19.4 (11.8–26.9)	0.782

Data are presented as medians and interquartile ranges. Differences between groups were analyzed via Kruskal-Wallis test; *p* values < 0.05 were considered to represent statistically significant differences. Apnea Hypopnea Index (AHI), Obstructive Apnea Index (OAI), oxygen saturation (SaO_2_), Central Apnea Index (CAI), Obstructive Desaturation Index (ODI).

## Data Availability

Not applicable.

## Supplementary Materials

The supporting information can be downloaded at: https://www.mdpi.com/article/10.3390/ijms241914674/s1.
