# Supplementary material for: Adipokines and Inflammatory Markers in Acute Myocardial Infarction Patients with and without Obstructive Sleep Apnea: A Comparative Analysis
Source: Int J Mol Sci. 2023 Sep 28;24(19):14674. doi: 10.3390/ijms241914674 (PMC10572714; doi:10.3390/ijms241914674)
Supplement: Supplementary file 1 [file ijms-24-14674-s001.zip › ijms-2529385-supplementary.pdf]

Supplementary Materials Table S1. Value of each variable in figures for future meta-analysis.

**Inflammatory marker**	**Control w/o OSA**	**AMI w/o OSA**	**Control w OSA**	**AMI w OSA**	**p-Value**
Adiponectin	10.94±2.97	9.14±5.21	8.86±3.06	11.71±7.13	0.391
Leptin	9.46±9.98	9.20±10.5	21.00±16.18	24.13±34.11	0.046
Resistin	3.69±1.27	7.40±3.43	4.25±1.73	7.15±4.20	0.003
IL-6	1.35±1.17	8.80±11.16	2.48±1.40	8.94±9.16	0.001
TNF-α	10.30±4.25	11.35±3.12	11.06±2.29	12.13±4.85	0.410
PAI-1	3.52±1.51	2.37±0.96	3.85±1.35	3.91±1.94	0.024
ET-1	1.83±0.35	3.31±1.42	2.15±0.45	3.22±2.10	<0.001
SAA	50.64±39.85	339±335.4	40.56±37.47	181.3±227.9	0.011
CRP	23.42±3.79	30.0±3.41	26.68±1.54	26.5±2.69	<0.001
